# Identification, Expression Analysis of the Hsf Family, and Characterization of Class A4 in *Sedum Alfredii* Hance under Cadmium Stress

**DOI:** 10.3390/ijms19041216

**Published:** 2018-04-17

**Authors:** Shuang-Shuang Chen, Jing Jiang, Xiao-Jiao Han, Yun-Xing Zhang, Ren-Ying Zhuo

**Affiliations:** 1 State Key Laboratory of Tree Genetics and Breeding, Chinese Academy of Forestry, Beijing 100091, China; chenshuang7876@sina.com (S.-S.C.); jiang2707@163.com (J.J.); hanxj@caf.ac.cn (X.-J.H.); yunxingzh78@126.com (Y.-X.Z.); 2 Key Laboratory of Tree Breeding of Zhejiang Province, The Research Institute of Subtropical of Forestry, Chinese Academy of Forestry, Hangzhou 311400, China

**Keywords:** *Sedum alfredii* Hance, heat shock transcription factor (Hsf), cadmium stress, class A4

## Abstract

*Sedum alfredii* Hance, a cadmium (Cd)/zinc (Zn)/lead (Pb) co-hyperaccumulating species, is a promising phytoremediation candidate because it accumulates substantial amounts of heavy metal ions without showing any obvious signs of poisoning. The heat shock transcription factor (Hsf) family plays crucial roles in plant growth, development, and stress responses. Although the roles of some Hsfs in abiotic stress have been well studied in model plants, the Hsf family has not been systematically investigated in heavy metal hyperaccumulators. Here, we comprehensively analyzed the *Hsf* gene family in *S. alfredii* based on a transcriptome under Cd stress. There were 22 *Hsf* members that were identified and phylogenetically clustered into three classes, namely, SaHsfA, SaHsfB, and SaHsfC. All of the three classes shared similar motifs. The expression profiles of the 22 *Hsf* members showed significant differences: 18 *SaHsfs* were responsive to Cd stress, as were multiple *SaHsp* genes, including *SaHsp18.1*, *SaHsp22*, *SaHsp26.5*, *SaHsp70*, *SaHsp90,* and *SaHsp101*. Two class A4 members, *SaHsfA4a* and *SaHsfA4c*, exhibited transcriptional activation activities. Overexpression of *SaHsfA4a* and *SaHsfA4c* in transgenic yeast indicated an improved tolerance to Cd stress and Cd accumulation. Our results suggest *SaHsfs* play important regulatory roles in heavy metal stress responses, and provide a reference for further studies on the mechanism of heavy metal stress regulation by *SaHsfs*.

## 1. Introduction

Abiotic stresses, such as heavy metal, heat, cold, drought, and salinity, adversely affect the growth and development of plants. They are defined by their negative impact on organisms in a given environment. Unlike animals, plants are sessile organisms. Therefore, they have had to develop defenses or adaptation mechanisms to deal with various stresses present during evolution [1,2,3,4]. These mechanisms can be processed including multiple genes and signaling pathways, which produce a series of physiological and biochemical changes in order to resist stress damage [5,6,7]. Each stage of these processes involves different types of transcription factors and *cis*-acting elements in stress-responsive promoters, which are controlled by different signal conditioning mechanisms in order to enable plant adaptation to environmental stresses [8]. Among these important transcription factors, heat shock transcription factors (Hsfs) are well known for responding to external high-temperature stress and activating the expression of heat shock proteins (Hsps) by combining with the heat shock element (HSE) in the thermal signal transduction pathway [2,4,7].

Hsfs are part of an evolutionarily conserved gene family belonging to eukaryotes [9,10]. The structure and function of Hsfs are considerably conserved, despite the fact that there are only a few genes that undergo biological genome replication events, from the lower organism to the higher living of the large genome [10,11]. Almost all Hsfs have a conserved DNA-binding domain (DBD) containing three helices and four sheets. This domain, with hydrophobic amino acids, can form a special structure, activating the formation of Hsfs to bind with the HSE element of *Hsp* genes’ promoter efficiently [7,10,12]. Similar to the DBD domain, the oligomerization domain (OD: HR-A/B regions) is a conserved domain located at the N-terminus of the Hsfs. Additionally, there are four other conserved structures, namely, the nuclear localization signal (NLS), nuclear export signal (NES), activator motif (AHA), and repression domain (RD). The *Hsf* gene family is divided into three classes based on the length of the flexible linker peptide between the DBD and HR-A/B, and the number of amino acids inserted between the HR-A and HR-B regions. Class A and B members contain AHA and RD motifs, respectively [7].

*Hsf* gene families are complex and large in higher plants. The size of the *Hsf* gene family varies greatly in the different plant species presented, with 21 members in the model plant *Arabidopsis thaliana*, 25 members in *Oryza sativa*, 28 members in *Populus trichocarpa*, 16 members in *Medicago truncatula*, 26 members in *Glycine max*, 25 members in *Zea mays*, and up to 56 members in *Triticum aestivum* [13,14,15,16,17,18,19,20,21]. Recently, many studies have demonstrated that the functions and characteristics of *Hsfs* differ among plant species in response to heat stress and other abiotic stresses [2,3,17,22,23,24]. Presently, two *HsfA* genes that are involved in cadmium (Cd) tolerance have been functionally characterized. *HsfA1a* from tomato (*Solanum lycopersicum* L. cv Ailsa Craig) induces an increase of melatonin levels under cadmium stress [25]. The *HsfA4a* gene in wheat (*Triticum aestivum*) and *O. sativa* has been reported to confer strong Cd tolerance [26]. These findings demonstrate the potential for research into heavy metal stress responses in the *Hsf* gene family.

*Sedum alfredii* Hance, a Cd/zinc (Zn)/lead (Pb) co-hyperaccumulating species of *Crassulaceae*, was first discovered in an abandoned Pb/Zn mine in Southeast China [27]. *S. alfredii*, a good material with perennial, asexual reproduction and a considerable biomass, can accumulate substantial amounts of heavy metal ions without displaying any obvious signs of poisoning. Thus, it is considered a promising candidate material in the field of phytoremediation. To date, several important physiological indices, including the accumulation capacity and translocation rates of different metal ions, have been characterized in this plant [28,29,30]. Additionally, some studies have been carried out on the molecular mechanism underlying the hyper-accumulation and hyper-tolerance to heavy metals in *S. alfredii*. Overexpression of the genes *SaMT2*, *SaCu/Zn SOD*, and *SaREF*, isolated from *S. alfredii*, conferred a higher tolerance to Cd stress in transgenic tobacco or *A. thaliana* [31,32,33]. Nevertheless, the Hsf family in *S. alfredii* has not yet been systematically investigated through phylogenetic, gene structure, conserved motif, and expression profiling analyses. Fortunately, the transcriptome data of *S. alfredii* were obtained [34], enabling the characterization of Hsf family members as well as their responses to heavy metal stress at the molecular level. In this study, bioinformatics and gene expression analyses were used to identify the 22 Hsf family members in *S. alfredii*. We also performed the conserved domain, motif, and phylogenetic analyses. Additionally, the expression patterns of the *SaHsf* genes were profiled in response to heavy metal stress. Finally, the overexpression of two SaHsfA4 members was shown to enhance Cd tolerance in yeast. These results provide a foundation for further functional research on *Hsf* genes in *S. alfredii* and may serve as a reference for future studies on the mechanism of heavy metal stress regulation by *SaHsfs*.

## 2. Results

### 2.1. Twenty Two SaHsf Members were Identified and Classified into Three Classes

To identify the *Hsf* genes in *S. alfredii*, a Hidden Markov Model (HMM) profile of the Hsf DBD domain (Pfam: PF00447) was used as a query against the *S. alfredii* transcriptome database. A total of 22 UniGenes containing DBD domains were obtained and annotated according to the Hsfs that were reported in other plants. Subsequently, the full coding regions of the candidate genes were cloned and sequenced. Finally, all of the *SaHsf* genes were verified using the online tools SMART and Pfam. Thereafter, the 22 *Hsf* genes in *S. alfredii* were classified and named according to the rules of the Hsf families from *A. thaliana* and *O. sativa* and the HEATSTER tool (Table 1). The coding sequence (CDS) size for the *SaHsfs* ranged from 849 to 1563 bp, and the corresponding predicted protein lengths and molecular weights ranged from 282 to 520 amino acids and 31.99 to 57.42 kDa (kilodalton), respectively (Table 1).

### 2.2. Phylogenetic Analysis of Hsfs in S. alfredii

**Figure 1:**
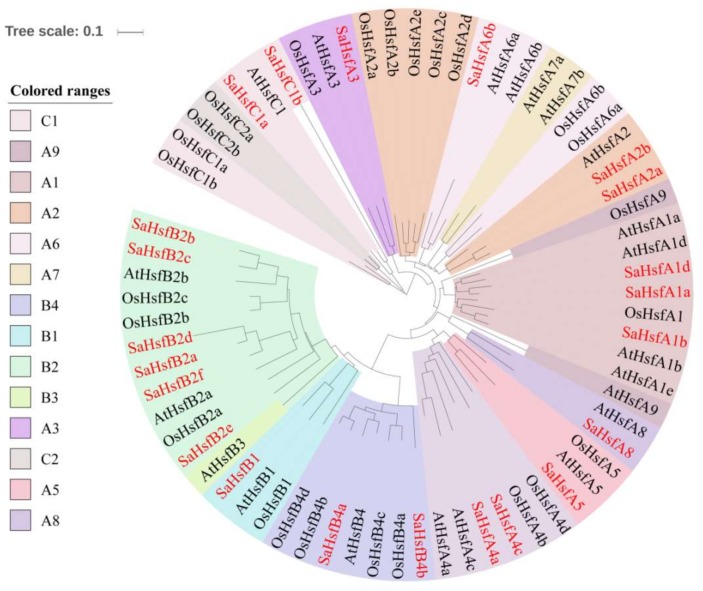
Phylogeny and distribution of heat shock transcription factor (Hsf) proteins. Phylogenetic tree of Hsf proteins from *A. thaliana*, *O. sativa*, and *S.alfredii*. The tree was generated with MEGA 6.0 software using the neighbor-joining (NJ) method. Hsfs in *S.alfredii* were labeled with the red color.

To examine the evolutionary characteristics of the Hsf proteins, a phylogenetic tree was constructed using the Hsf protein sequences from *S. alfredii* and the model plants *A. thaliana* and *O. sativa*. The full-length amino acid sequences of these Hsf proteins are shown in Supplementary File 1 and Table S1. According to the phylogenetic tree, the SaHsfs were grouped into three classes, namely, A, B, and C (Figure 1). Among them, class A was the largest subgroup with 11 Hsf proteins from seven subclasses (A1, A2, A3, A4, A5, A6, and A8), followed by class B with 9 members from three subclasses (B1, B2 and B4), while class C included only two members from subclass C1 (Figure 1, Table 2). Additionally, the class A members were divided into three smaller branches. Specially, the class A member SaHsfA3 had relatively distant evolutionary relationships with other class A members. Unlike class A3, the class A4 and A5 members had relatively close evolutionary relationships with class B members. No members were found in the A7 subclass and only one member was found in the B1 subclass, when we compared the numbers of each subclass in the SaHsf family with those in other plant species—such as *A. thaliana* [19], *O. sativa* [20], *P. trichocarpa* [18], and *Z. mays* [16] (Table 2). In addition, the B2 subclass included six members, while this subclass contained less than five members in other plant species (two members in *A. thaliana*, three members in *O. sativa* and *P. trichocarpa*, and four members in *Z. mays*).

### 2.3. Conserved Domains and Motifs in SaHsf Proteins

**Figure 2:**
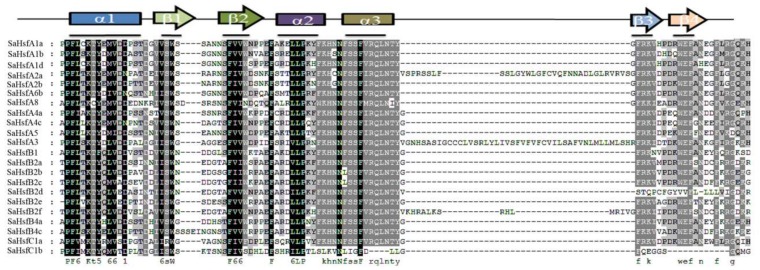
Multiple sequence alignment of the DNA-binding domain (DBD) of the Hsf protein family in *S. alfredii*. The different backgrouds, black and gray, indicated completely and partly conserved amino acids in proteins, respectively.

**Figure 3:**
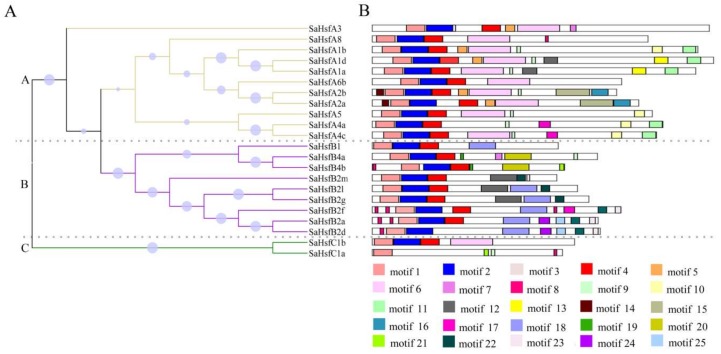
Phylogenetic relationships and motif compositions of the Hsf family in *S. alfredii*: (**A**) multiple alignment of Hsf proteins from *S. alfredii* was performed using MEGA 6.0 by the neighbor-joining (NJ) method with 1000 bootstrap replicates, which represented by circles; (**B**) schematic representation of each of the conserved motifs in the Hsf proteins was identified by the MEME online tool. Different motifs are represented by different colored boxes. The dashed lines are used to cluster and distinguish the genes into the A, B, and C classes.

The detailed knowledge available on the Hsf functional domains in the model plants *A. thaliana* and *O. sativa* enabled us to analyze the domains of the 22 SaHsfs. Six conserved domains, including the DBD, HR-A/B, NLS, AHA, RD, and NES, were predicted in the *SaHsf* family using the online tool HEATSTER (Table 3). The most conserved domains (DBD and HR-A/B) in Hsfs existed in all of the predicted SaHsf proteins. The DBD domain consisted of three helices and four sheets (Figure 2). However, SaHsfB2d and SaHsfC1b had no β 3 and β 4 sheets. Most of the SaHsf proteins included the NLS domain, except for SaHsfB1 and SaHsfB2m. The NES domain was detected in two A subclasses (A1 and A4 members) and one B subclass members (SaHsfB4a). With the exception of SaHsfA1a and SaHsfA1d, all of the other class A members had AHA domains. However, this domain was not found in class B or C. All of the class B members had the RD domain, except for SaHsfB2e. The online tool MEME was used to search for motifs in the SaHsf proteins. There were 25 potential motifs that were found, the details of these motifs are given in Table 4. As shown in Figure 3B, motifs 1, 2, and 4 (inferred as DBD domain) were found in all of the 22 SaHsf members, however, motifs 2 and 4 were missing in SaHsfC1b. Different subclasses contained the same motifs and had their own unique motifs. In subclass B, motif 22 was only found in SaHsfB2 members, while motif 18 was found in SaHsfB1 and SaHsfB2 members, and motifs 19 and 20 were only discovered in SaHsfB4 members. Similarly, motifs 7, 9, and 11 were unique to SaHsfA members, while motifs 15 and 16 existed only in SaHsfA2 members. Moreover, SaHsfC1a contained motif 6, which was also found in class A members. Notably, motif 3 was inserted between motif 2 and motif 4 in SaHsfA3, and motif 7 was also unique to this protein. As shown in Figure 3A, SaHsfA3 had a relatively distant relationship with other class A members. This result might have been related to the protein motifs. Generally, most of the closely related members had similar motif compositions.

### 2.4. Expression Profiles of SaHsfs

**Figure 4:**
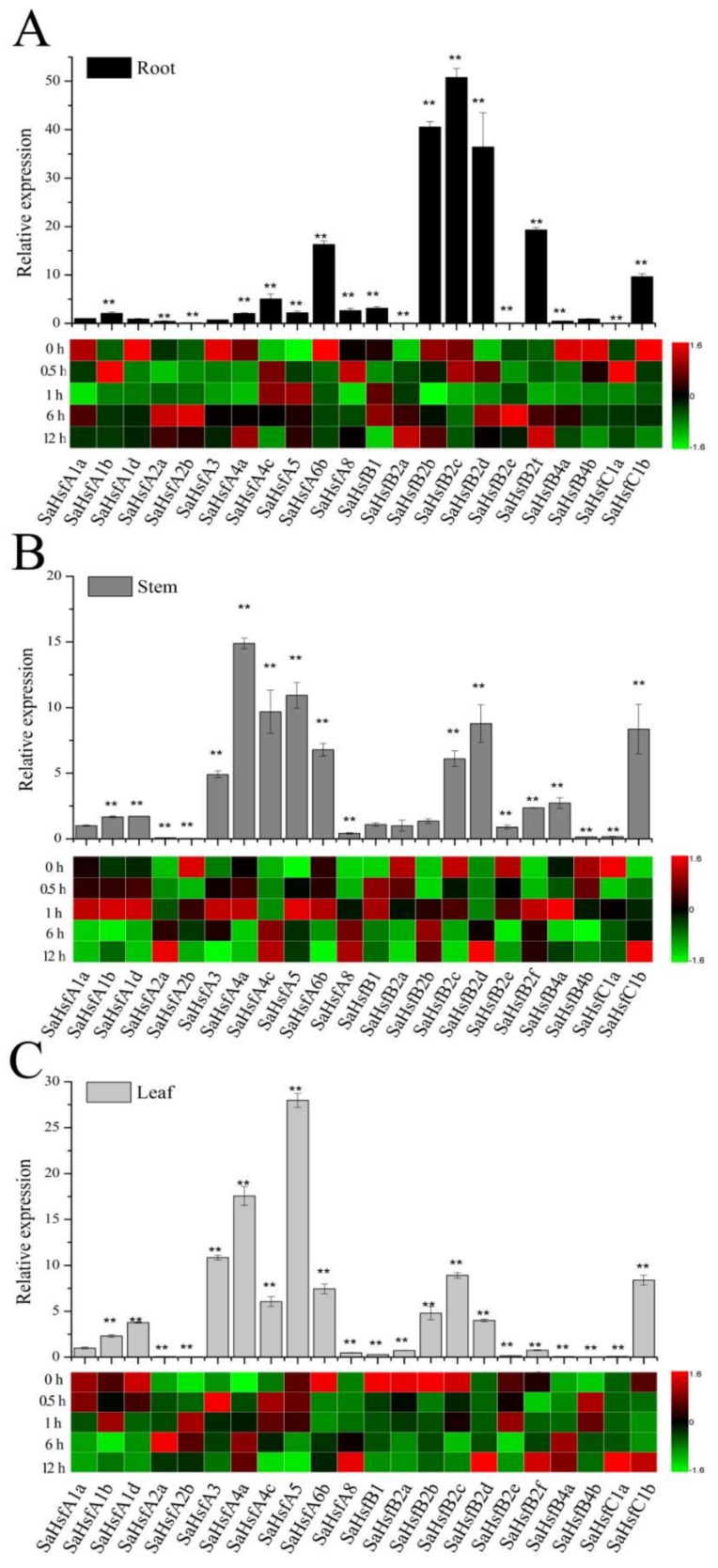
Expression profiles of *SaHsfs* in different tissues under normal and cadmium (Cd) stress conditions: (**A**) root; (**B**) stem; (**C**) leaf. Column chart and heat map representation for the expression patterns of the 22 *SaHsf* genes under normal and CdCl_2_ treatment, respectively. The normalized mRNA levels of *SaHsfA1a* (y-axis “Relative mRNA expression”) were set arbitrarily to 1 in the column chart, under normal condition. The normalized mRNA levels without treatment were set arbitrarily to 1 in the heat map. Different colors correspond to log2 transformed values compared with the control (0 h). Green and red represent the low and high level of transcript abundance, respectively. Bars indicate means ± standard deviations (SDs) of at least three independent biological experiments. **—*p* < 0.01.

To investigate the potential functions of the *SaHsf* genes under normal conditions, expression profiles of the SaHsf family members in *S. alfredii* were generated using quantitative real-time polymerase chain reaction (qRT-PCR) data from three tissues, including root, stem, and leaf (Figure 4). The expression patterns of most of the *SaHsf* genes were similar in different tissues. *SaHsfA1b*, *SaHsfA4a*, *SaHsfA4c*, *SaHsfA5, SaHsfA6b*, *SaHsfB2c*, *SaHsfB2d*, and *SaHsfC1b* were constitutively expressed at relatively high levels, while *SaHsfA2a*, *SaHsfA2b*, *SaHsfB2a*, *SaHsfB2e*, *SaHsfB4b*, and *SaHsfC1a* were expressed at low levels in all of the tested tissues. Some genes were expressed greatly in specific tissues. For example, the relative expression levels of *SaHsfB2f* in the root and *SaHsfB4a* in the stem were higher than in other tissues. In addition, *SaHsfA8* and *SaHsfB1* were detected only in the root, while *SaHsfB4a* was only expressed in the stem (Figure 4).

To examine the heavy metal response patterns of the *SaHsfs* in *S. alfredii*, we determined their expression levels in different tissues (root, stem, and leaf) under CdCl_2_ treatment. As a result of the properties of the experimental material (a Cd/Zn/Pb co-hyperaccumulating species), 11, 15, and 12 *SaHsf* genes were responsive to heavy metal (Cd) stress in the root, stem, and leaf, respectively (Figure 4). As shown in Figure 4A, *SaHsfA2a*, *SaHsfA2b*, *SaHsfA5*, *SaHsfB2a*, *SaHsfB2e*, and *SaHsfC1a* were up-regulated under CdCl_2_ treatment in the root. Conversely, four members, *SaHsfA6b*, *SaHsfB2b*, *SaHsfB2c*, and *SaHsfC1b*, were down-regulated compared with their expression levels under normal conditions. Other genes, *SaHsfA1b*, *SaHsfA4c*, *SaHsfA8*, *SaHsfB2d*, and *SaHsfB2f*, which had high expression levels under control conditions, were also up-regulated under CdCl_2_ treatment. A similar phenomenon was also found in the stem and leaf (Figure 4B,C). Specifically, *SaHsfB2a* and *SaHsfB2e* were only up-regulated by Cd stress in the root, as were *SaHsfA1a*, *SaHsfA1d*, *SaHsfB1*, and *SaHsfC1b* in the stem, and *SaHsfB4b* in the leaf. Unlike these genes, *SaHsfA1b*, *SaHsfA4a*, *SaHsfA4c*, *SaHsfA8*, and *SaHsfB2f* were up-regulated in all of the tested tissues.

Among the up-regulated genes, *SaHsfA5* was strongly induced in the root and stem at 1 h after Cd treatment, while its expression was down-regulated in the leaf. Notably, *SaHsfA4a* was induced at the earlier stage of treatment (0.5–1 h) in the stem, and at the late stage (6–12 h) in the root and leaf, while *SaHsfA4c*, in the same class, had an opposite expression pattern.

### 2.5. SaHsfs and Their Downstream Genes, SaHsps, Exhibited Similar Expression Patterns

**Figure 5:**
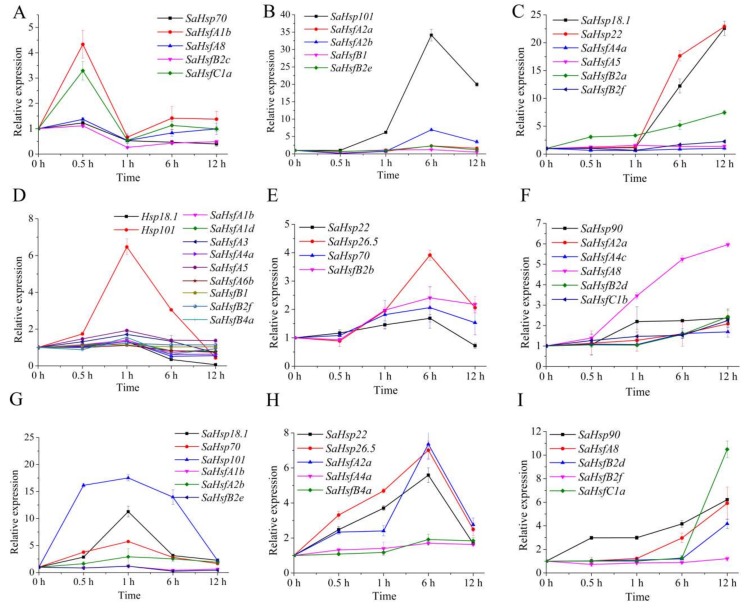
The expression profiles of *SaHsps* and *SaHsfs* in different tissues under Cd stress: The normalized mRNA levels without treatment were set arbitrarily to 1. (**A**–**C**) three similar expression patterns between *SaHsps* and *SaHsfs* in the root; (**D**–**F**) three similar expression patterns between *SaHsps* and *SaHsfs* in the stem; (**G**–**I**) three similar expression patterns between *SaHsps* and *SaHsfs* in the leaf. Bars indicate means ± standard deviations (SDs) of at least three independent biological experiments.

The expression patterns of the *SaHsps*, as downstream genes of *SaHsfs*, were tested and compared with those of the *SaHsfs* using qRT-PCR. There were three different expression profiles that were observed among the *SaHsfs* and *SaHsps* (Figure 5). At the earlier stage of the Cd treatment (0.5 - 1 h), *SaHsp70* was up-regulated in the root together with *SaHsfA1b*, *SaHsfA8*, *SaHsfB2c*, and *SaHsfC1a*, while *SaHsfA1b*, *SaHsfA2b*, and *SaHsfB2e* were up-regulated in the leaf, along with *SaHsp18.1* and *SaHsp101* (Figure 5A,G). There were 10 *SaHsf* genes (including *SaHsfA1*, *SaHsfA3*, *SaHsfA4a*, *SaHsfA5*, *SaHsfA6b*, *SaHsfB1*, *SaHsfB2f*, and *SaHsfB4a*) that had similar expression patterns to *SaHsp18.1* and *SaHsp101*, with a peak in the stem after 1 h of Cd treatment (Figure 5D). After 6 h of treatment, *SaHsp101*, *SaHsp22*/*SaHsp26.5/SaHsp70*, and *SaHsp22*/*SaHsp26.5* reached the highest expression levels in the root, stem, and leaf, respectively. Similar expression profiles were also found among other *SaHsfs*, namely, *SaHsfA2a*, *SaHsfA2b*, *SaHsfA4a*, *SaHsfB1*, *SaHsfB2b*, *SaHsfB2e*, and *SaHsfB4a* (Figure 5B,E,H). A continuously increasing expression pattern was considered to be the third profile in the root, stem, and leaf (Figure 5C,F,I). *SaHsfA4a*, *SaHsfA4c*, *SaHsfA5*, *SaHsfA8*, *SaHsfB2a*, *SaHsfB2d*, *SaHsfB2f*, *SaHsfC1a*, and *SaHsfC1b* indicated this expression pattern, as did the *SaHsp18.1*, *SaHsp22*, and *SaHsp90* genes (Figure 5C,F,I). In addition, the *SaHsp* genes exhibited higher expression levels than the *SaHsf* genes in *S. alfredii* under Cd stress (Figure 5).

### 2.6. Class SaHsfA4’ Expression Enhanced Cd Tolerance in Yeast

**Figure 6:**
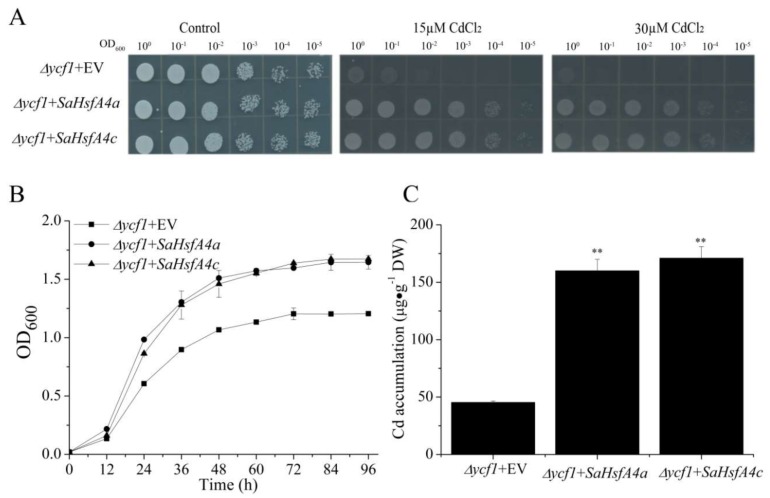
Overexpression of *SaHsfA4a* or *SaHsfA4c* increases the Cd tolerance and accumulation in yeast. (**A**) the growth of ∆*ycf1* yeast mutants transformed with the empty vector pYES2.0 or with pYES-DEST52 harboring *SaHsfA4a* or *SaHsfA4c*; (**B**) time-dependent growth of yeast strains in synthetic-galactose-uracil (SG-U) liquid medium supplemented with 15 μM CdCl_2_; and (**C**) the accumulation of Cd in ∆*ycf1* yeast cells. Bars indicate means ± standard deviations (SDs) of at least three independent biological experiments. Two asterisks indicate a significant difference at *p* <0.01 from the ∆*ycf1* + EV.

To analyze the function of the class *SaHsfA4* members (*SaHsfA4a and SaHsfA4c*), the genes were expressed in a *Saccharomyces cerevisiae* yeast mutant strain (∆*ycf1*) that was susceptible to excessive Cd. The verified recombinant plasmids (pYES-DEST52-*SaHsfA4a* and pYES-DEST52-*SaHsfA4c*) and empty vector (pYES2.0) were transformed into ∆*ycf1* cells, which were then grown in a synthetic-galactose-uracil (SG-U) medium. *∆ycf1* cells expressing *SaHsfA4a* and *SaHsfA4c* exhibited remarkably enhanced growth status when compared with *∆ycf1* cells transformed with the empty vector (*∆ycf1* + EV) on SG-U medium with CdCl_2_ (Figure 6A,B). Additionally, the metal content was measured in the yeast cells expressing *SaHsfA4a*, *SaHsfA4c*, or the empty vector, which were grown in the presence of Cd for about 96 h. A significantly increased accumulation of Cd was observed in the yeast cells expressing *SaHsfA4a* or *SaHsfA4c* compared with the control (Figure 6C). Furthermore, yeast cells (AH109) containing either the control (pGBKT7) or fusion plasmids (pGBKT7-*SaHsfA4a* and pGBKT7-*SaHsfA4c*) grew well on SD/Trp^−^ plates. Only the yeast cells containing fusion plasmids could grow on SD/Trp^−^His^−^ plates and they turned blue in the presence of X-α-gal (Supplementary Figure S1). These results suggested that *SaHsfA4a* and *SaHsfA4c* were transcription activators and could activate the expression of the *GAL4* upstream activation sequence-driven *LacZ* reporter gene.

## 3. Discussion

The Hsf family has been researched in many plant species [2,21,35,36,37], but little information is available on the characteristics of the *Hsfs* in *S. alfredii* under heavy metal stress. In this study, 22 *Hsf* genes were screened and identified in *S. alfredii*, based on transcriptome data (Table 1). The phylogenetic analysis indicated that the *SaHsfs* could be divided into three major groups corresponding with those in *A. thaliana* and *O. sativa* (Figure 1), which was consistent with previous reports [13,14,15,18]. Although the total number of *SaHsf* genes was similar to those in other plant species, such as *A. thaliana*, *O. sativa*, *P. trichocarpa*, and *Z. mays* (Table 2), the sizes of some subclasses in *S. alfredii* differed from those in other species. For instance, no subclass A7 and A9 members were found. Conversely, the number of genes in class B2 was greater than those in other species, suggesting that gene loss and gene duplication events had occurred at the different stages of the evolutionary process, resulting in Hsf diversity [13,38]. Each class of *SaHsfs* shared similar motifs (Figure 2), implying that the *Hsf* genes were evolutionarily well conserved or had similar regulatory functions in *S. alfredii*.

Previous studies on Hsfs had focused on their roles in responses to abiotic stresses such as heat, salt, drought, cold, and hormones [3,12,17,23,24,39]. Only a few reports had indicated that some Hsf members played critical roles in enhancing Cd tolerance [25,26]. Here, a single genotype of *S. alfredii* seedlings was asexually propagated and used in CdCl_2_ treatment. We examined the expression profiles of the *SaHsf* genes in different tissues after 400 μM CdCl_2_ treatment. More than half of the *SaHsf* genes were responsive to heavy metal (Cd) stress, which may be due to the properties of the experimental material (*S. alfredii*) as a Cd/Zn/Pb co-hyperaccumulator. Under normal conditions, the genes *SaHsfA1b*, *SaHsfA4a*, *SaHsfA4c*, *SaHsfA5*, *SaHsfA6b*, *SaHsfB2c*, *SaHsfB2d*, and *SaHsfC1b* were expressed in all tested tissues, while *SaHsfA8, SaHsfB1*, and *SaHsfB2f* transcripts were detected primarily in the root and *SaHsfB4a* transcripts in the stem (Figure 4), suggesting that the expression of these Hsf family members is tissue specific. After CdCl_2_ treatment, *SaHsfB2a* and *SaHsfB2e* were only up-regulated in the root, *SaHsfA1a*, *A1d*, *B1* and *C1b* in the stem, and *SaHsfB4b* in the leaf. These results suggested that the Hsf family members in *S. alfredii* are induced in a tissue-specific manner under Cd stress. It was reported that overexpression of *HsfA1a* from *S. lycopersicum* L. cv Ailsa Craig enhanced plant tolerance to Cd [25], and that class HsfA4 genes in wheat and rice conferred Cd tolerance [26]. Our study found that *SaHsfA1b*, *SaHsfA4a*, and *SaHsfA4c* were up-regulated in all of the tested tissues under Cd stress. We also found that *SaHsfA4a* and *SaHsfA4c* had the opposite expression patterns at different stages of treatment in different tissues. *SaHsfA5* expression was strongly induced in the root and stem, and reduced in the leaf under Cd treatment. In *Arabidopsis*, *AtHsfA5* acted as a specific repressor of *AtHsfA4* and was considered to be an antiapoptotic factor [9,40]. Furthermore, HsfA4 and HsfA5 were found to have a close relationship in the phylogenetic tree and had similar motifs in their C terminal [40]. In our study, a similar result was observed in *S. alfredii* (Figure 2). However, the expression profiles of *HsfA4* and *HsfA5* were very different. Thus, their roles in heavy metal response deserve further investigation.

*Hsps* are the downstream genes of *Hsfs* that protect plants against abiotic stress damage [40,41]. *Hsfs* are responsible for the transcription of *Hsps*, such as small heat shock proteins (*sHsps*), *Hsp70*, and *Hsp101* [7,40,42]. *Hsps* could be induced by various abiotic stresses, such as cold, heat, and salt [7,42,43,44]. Overexpression of *GmHsfA1* could enhance heat tolerance by regulating downstream *Hsp* genes (such as *GmHsp22* and *GmHsp70*) in transgenic soybeans [45]. It was also reported that multiple *Hsp* genes, including *Hsp18.1*, *Hsp22*, *Hsp26.5*, *Hsp70*, and *Hsp101*, were positively induced by heat treatment in the transgenic *Arabidopsis* and tall fescue overexpressing *FaHsfA2c* [42]. *CmHsp70* and *CmHsp90* were considered the direct target genes of *CmHsfA4* in the transgenic *Chrysanthemum* plants, both in non-stress and salt stress conditions. In our study, all of the tested *Hsp* genes, including *SaHsp18.1*, *SaHsp22*, *SaHsp26.5*, *SaHsp70*, *SaHsp90*, and *SaHsp101*, were up-regulated remarkably by Cd stress in the root, stem, or leaf (Figure 5). Moreover, three similar expression patterns between *SaHsps* and *SaHsfs* were exhibited under Cd treatment. These results suggested that *SaHsfs* could regulate the expression levels of *SaHsps* when the plants were exposed to heavy metal stresses.

Heterologous expression of the class A4 Hsfs (*SaHsfA4a* and *SaHsfA4c*) conferred Cd tolerance to yeast (Figure 6). Previously, class A4 Hsfs had been reported as being induced by other abiotic stresses in plants. *AtHsfA4a* had been involved in high-light and oxidative stress responses by regulating the transcription of *APX1* and *ZAT12* genes [44,46]. Co-overexpression of *Helianthus annuus HaHSFA4a* and *HaHSFA9* enhanced tolerance to dehydration and severe oxidative stress in transgenic tobacco [47]. Overexpression of the *CmHsfA4* gene positively enhanced salt stress tolerance in transgenic *Chrysanthemum* [44]. Additionally, studies have shown that *OsHsfA4a and TaHsfA4a* conferred tolerance to Cd [26]. Here, the *∆ycf1* yeast strain harboring the fusion vectors (pYES-DEST52-*SaHsfA4a* or pYES-DEST52-*SaHsfA4a*) exhibited better growth than the control (*∆ycf1 +* EV) (Figure 6A,B). Moreover, the transgenic yeast harboring the fusion vectors had a higher Cd content than the control (Figure 6C). These results indicated that the class *SaHsfA4* members might have been involved in responding to the Cd stress, and that these genes could complement the Cd sensitivity in the mutant yeast strain. Additionally, transcriptional activation assay revealed SaHsfA4a and SaHsfA4c were transcription activators in yeast cells (Supplementary Figure S1). Thus, we speculated that *SaHsfA4a* and *SaHsfA4c* as transcription activators, could activate target genes to enhance Cd tolerance and improve Cd accumulation. Similarly, Shim et al. found that transgenic yeast expressing *OsHsfA4a* and *TaHsfA4a* were tolerant to Cd, and that the yeast strain overexpressing *TaHsfA4a* grew better in liquid cultures supplemented with CdCl_2_ than the control [26].

Overall, we have presented a comprehensive analysis of the *Hsf* gene family members in *S. alfredii* as well as their expression under Cd stress. The 22 Hsf members were phylogenetically clustered into three classes. The expression profiles of the *SaHsfs* showed significant differences; among them, 18 *SaHsfs* were found to respond to Cd stress. *SaHsfA4a* and *SaHsfA4c* exhibited transcriptional activation activities. Overexpression of the *SaHsfA4a* or *SaHsfA4c* gene enhanced tolerance to Cd stress in transgenic yeast. Our results will be beneficial for elucidating the mechanism of heavy metal stresses regulation by *SaHsfs*.

## 4. Methods

### 4.1. Plant Materials and Stress Treatments

A hyperaccumulating ecotype of *S. alfredii*, collected from an old Pb/Zn mine in Quzhou City, Zhejiang Province, China, was first identified by Yang et al. [27].

The seedlings were water-cultivated in an artificial climate chamber for long-day treatment (16 h light/8 h dark cycle) at 25 °C. The *S. alfredii* seedlings from a single genotype were asexually propagated and grown in a half-strength Hoagland solution for about three weeks. For heavy metal stress treatment, the roots were dipped into a solution containing 400 μM CdCl_2_, while a set of control seedlings were similarly cultured in a half-strength Hoagland solution. The treated root, stem, and leaf were then harvested at 0, 0.5, 1, 6, and 12 h. All of the samples from the three biological replicates were frozen at −80 °C for subsequent analysis.

### 4.2. Identification of SaHsfs in S. alfredii

The coding and peptide sequences of candidate *SaHsfs* were searched in the transcriptome database of *S. alfredii* [34]. An HMM search (http://www.ebi.ac.uk/Tools/hmmer/search/hmmsearch) was conducted to identify and retrieve possible Hsfs containing DBD (Pfam: PF00447) domains in *S. alfredii*. Some UniGenes that were produced by the transcriptome sequencing did not contain the full coding regions of the genes. Thus, the putative *S. alfredii SaHsfs* with incomplete coding sequences were conducted and cloned to obtain the full coding regions through a homologous cloning strategy. The Hsf homologs in *A. thaliana* and *O. sativa* were obtained from TAIR (https://www.arabidopsis.org/) and the TIGR-Rice Genome Annotation Project (http://rice.plantbiology.msu.edu/), respectively (Supplementary Table S1). All of the candidate genes were confirmed by SMART (http://smart.embl-heidelberg.de/) and Pfam (http://pfam.xfam.org/search). In addition, all of the *SaHsf* genes were named according to the orthologous genes in *A. thaliana*. The molecular weights (kDa) and isoelectric points (pI) of the Hsfs were calculated by the ExPASy program (http://web.expasy.org/compute_pi) and DNAMAN software.

### 4.3. Multiple Sequence Alignment, Conserved Motif and Domain Prediction

ClustalX was used to align the amino acid sequences of all of the candidate *S. alfredii* Hsf proteins. Subsequently, the result was edited manually with GeneDoc. MEME (http://meme.sdsc.edu) was used to identify the motifs in the candidate sequences and was run locally with the following parameters: number of repetitions = any; maximum number of motifs = 25; and optimum motif width = 6–100 residues. The distribution diagram of the motifs was edited with the IBS software [48]. HEATSTER (http://www.cibiv.at/services/hsf/) online tools were used to analyze the SaHsf proteins’ typical functional structural domains [7].

### 4.4. Phylogenetic Analysis and Classification of SaHsf Genes

The amino acid sequences of the Hsf proteins from *S. alfredii*, *Arabidopsis* and *rice* were aligned using the ClustalX program. Subsequently, the MEGA 6.0 software was used to construct an unrooted neighbor-joining phylogenetic tree with a bootstrap test that was replicated 1000 times [49]. The *SaHsf* genes were assigned to different groups based on the phylogenetic tree and the HEATSTER predictions.

### 4.5. Total RNA Isolation and Expression Analysis

The total RNA was isolated from root, stem, and leaf using a total RNA kit (NORGEN, Thorold, ON, Canada) and treated with DNase I (TaKaRa, Dalian, China) to digest any genomic DNA. First-strand cDNA was generated using PrimeScript™ RT Master Mix (TaKaRa, Dalian, China) following the manufacturer’s instructions and then stored at −20 °C until use.

qRT-PCR was carried out in triplicate on an Applied Biosystems 7300 Real-Time PCR System thermal cycler (Applied Biosystems, CA, USA) using SYBR^®^ Premix Ex Taq™ reagent, (TaKaRa, Dalian, China). The primer sequences that were used in qRT-PCR are shown in Supplementary Table S2. The amplification procedure was performed according to a previous study [33,50]. The relative expression level of each of the *SaHsf* genes was calculated based on the comparative threshold cycle (2^−ΔΔCT^) method. The *SaUBC9* gene was used as an endogenous reference gene to normalize the threshold values (Ct) of the *SaHsf* and *SaHsp* genes [51]. The primers were designed using the online tool, Primer 3.0 (http://bioinfo.ut.ee/primer3-0.4.0/primer3/), based on the non-conserved regions of the *SaHsf* and *SaHsp* genes, and were synthesized by BioSune Company (Shanghai, China).

The normalized mRNA levels of *SaHsfA1a* (y-axis “Relative mRNA expression”) were set arbitrarily to 1, under normal condition. To analyze the *SaHsf* genes’ relative expression under Cd stress, the normalized mRNA levels without treatment (y-axis “Relative mRNA expression”) were also set arbitrarily to 1. Subsequently, the z-score method was used to normalize the original expression levels of the *SaHsfs*, in order to draw a heat-map with HemI (The Cuckoo Workgroup, Wuhan, China). The absolute signal intensity ranged from −1.6 to +1.6, and the color scale (green and red) represented the expression values (low and high level). Bars indicated means ± standard deviations (SDs) of at least three of the independent biological experiments.

### 4.6. Heterologous Expression of SaHsfA4a and SaHsfA4c in Yeast

The specific primers *SaHsfA4a*-F/R and *SaHsfA4c*-F/R (Supplementary Table S2) were used to amplify the open reading frames in *S. alfredii*. The purified PCR products were then cloned into the Gateway entry vector pDONR222 (Invitrogen, Carlsbad, USA), and recombined into pYES-DEST52 to generate pYES-DEST52-*SaHsfA4a* and pYES-DEST52-*SaHsfA4c*, respectively. The ∆*ycf1* strain was transformed with each construct, using the lithium acetate method [33,52]. The empty pYES2.0 vector was used as control. To perform the Cd tolerance test, the transgenic yeast lines were grown up to OD_600_ = 1, and then serially diluted (OD_600_ = 10^0^, 10^−1^, 10^−2^, 10^−3^, 10^−4^, and 10^−5^), spotted on SG-U agar plates supplemented with 0, 15, and 30 µM CdCl_2_, and incubated at 28 °C for 3 days [33,50]. Additionally, the relative growth of transformants was determined by measuring OD_600_ at 12 h intervals. For the Cd-uptake assay, yeast cells that were transformed with the empty, pYES-DEST52-*SaHsfA4a* or pYES-DEST52-*SaHsfA4c* vector were grown at 28 °C on SG-U, supplemented with 15 µM CdCl_2_ for 96 h, and finally, the Cd accumulation was measured [50]. 

### 4.7. Transcriptional Activation Activity Assay of Two SaHsfA4 Members in Yeast

Transcriptional activation vectors were constructed and specific primers were designed (Supplementary Table S2) using a GBclonart Cloning Kit (GBI, Suzhou, China). The complete coding sequences of *SaHsfA4a* and *SaHsfA4c* were amplified by PCR using specific primers. The PCR products were cloned into the pGBKT7 vector to create pGBKT7-SaHsfA4a and pGBKT7-SaHsfA4c. The sequence-verified plasmids were transformed in the yeast strain AH109, using the lithium acetate method [52]. The transformed strains were confirmed by PCR and sequencing, and then plated on a SD/Trp^−^, SD/Trp^−^His^−^, or SD/Trp^−^His^−^ + X-α-gal medium. Transcription activation activity was evaluated according to the growth status of the yeast cells after incubating the plates at 28 °C for 3 days.

## 5. Conclusions

In the present study, a comprehensive analysis of the Hsf family was performed, including phylogenetic, conserved domain, and motif analyses and expression profiling, under Cd stress based on transcriptome sequencing. A total of 22 *Hsf* members were identified from *S. alfredii* using bioinformatics. They were phylogenetically clustered into three classes, namely, SaHsf*A*, *B*, and *C*, according to their structural and phylogenetic features. According to the phylogenetic tree, the majority of the subfamilies contained members from *Arabidopsis*, *rice*, and *S. alfredii*, suggesting that the functions of most Hsfs had been conserved during the evolutionary progress. The largest subgroup, class A, included 11 *Hsf* members, followed by class B with 9 members, while class C contained only two members. Each class shared similar motifs, indicating that the *Hsfs* were markedly conserved during the evolution of *S. alfredii*. In addition, expression analysis indicated that the *Hsfs* and *Hsps* in *S. alfredii* may play important roles in responses to Cd stress. Moreover, class *SaHsfA4* members exhibited transcriptional activation activities and positively regulated Cd stress tolerance and accumulation in yeast. Our results provide a solid foundation for further functional dissection of the *SaHsf* family, and will improve our understanding of the characteristics of *SaHsf* genes in hyperaccumulating species.

## Figures and Tables

**Table 1:** The basic information list of the identified heat shock transcription factors (Hsfs) in *S. alfredii*.

Number	Gene Name	ORF Length (bp)	No. of AA	Mol. Wt. (kDa)	pI
1	*SaHsfA1a*	1482	493	55.11	4.66
2	*SaHsfA1b*	1491	496	54.84	5.02
3	*SaHsfA1d*	1563	520	57.42	4.65
4	*SaHsfA2a*	1221	406	45.82	4.95
5	*SaHsfA2b*	1122	373	42.69	4.43
6	*SaHsfA3*	1545	514	57.75	5.04
7	*SaHsfA4a*	1332	443	50.10	5.36
8	*SaHsfA4c*	1302	433	49.32	6.20
9	*SaHsfA5*	1284	427	47.53	5.45
10	*SaHsfA6b*	1143	380	43.33	6.69
11	*SaHsfA8*	1263	420	48.63	4.89
12	*SaHsfB1*	855	284	31.83	4.50
13	*SaHsfB2a*	1038	345	37.49	6.91
14	*SaHsfB2b*	942	313	34.47	4.62
15	*SaHsfB2c*	993	330	36.52	4.99
16	*SaHsfB2d*	1047	348	37.61	5.35
17	*SaHsfB2e*	849	282	31.99	7.89
18	*SaHsfB2f*	1140	379	41.08	8.15
19	*SaHsfB4a*	1032	343	39.57	6.74
20	*SaHsfB4b*	885	294	33.81	7.47
21	*SaHsfC1a*	930	309	35.11	5.45
22	*SaHsfC1b*	873	290	32.64	6.73

Note: ORF—open reading frame; No. of AA—number of amino acids; Mol. Wt—molecular weight; pI—isoelectric point.

**Table 2:** Members of the Hsfs classes and subclasses in different plant species.

Class	Subclass	*A. thaliana*	*O. sativa*	*P. trichocarpa*	*Z. mays*	*S. alfredii*
A	A1	4	1	3	2	2
	A2	1	5	1	2	3
	A3	1	1	1	1	1
	A4	2	2	3	3	2
	A5	1	1	2	1	1
	A6	2	2	2	2	1
	A7	2	0	2	2	0
	A8	1	0	2	2	1
	A9	1	1	1	0	0
B	B1	1	1	1	2	1
	B2	2	3	3	4	6
	B3	1	0	2	0	0
	B4	1	4	4	1	2
C	C1	1	2	1	2	2
	C2	0	2	0	1	0
	Total members	21	25	28	25	22

**Table 3:** Function domains found by HEATSTER in SaHsfs.

Name	Domains					
	DBD	HR-A/B	NLS	AHA	RD	NES
SaHsfA1a	24–117	129–186	204–214 (RIIGENNKKRR)			454–461 (ITDQMELL)
SaHsfA1b	20–113	133–194	215–225 (RRITSSNKKRR)	(AHA2) 431–440 (DVFWEQFLST)		481–488 (LTSQMGLL)
SaHsfA1d	37–130	156–220	238–248 (RRINEANKKQR)			488–495 (ITDQIGLL)
SaHsfA2a	33–159	175–239	255–268 (RKALDGANVKRKRT)	(AHA1) 314–322 (SLLRAGLES)		
SaHsfA2b	25–118	134–198	214–228 (RKALDDAYSKRKRRL)	(AHA1) 313–320 (QMLWDELV)		
SaHsfA3	57–194	222–268	294–303 (KMKRKFITHH)	(AHA3) 472–481 (FTDGWEFGSM)		
				(AHA4) 489–495 (LELGSPS)		
SaHsfA4a	11–104	130–187	205–208 (KKRR)	(AHA1) 253–262 (ITHWEKIIYQ)		430–437 (LVEQMGHI)
				(AHA2) 383–392 (DTFWAQFLTE)		
SaHsfA4c	22–115	139–196	214–217 (KKRR)	(AHA1) 264–273 (FSYWENILYS)		420–427 (ITEQMGQL)
				(AHA2) 369–378 (DVFWEQYLTE)		
SaHsfA5	19–112	132–189	200–215 (TKINSMEFSAYSKKRR)	(AHA) 404–413 (DAFWEQYLTE)		
SaHsfA6b	48–141	162–227	250–257 (AAANKRRH)	(AHA) 332–341 (QVFWEGFLNN)		
SaHsfA8	13–121	154–211	326–334 (VDNTWYANH)	(AHA1) 312–321 (DDAILDHFIF)		
SaHsfB1	7–100	150–187			255–261 (KLFGVWL)	
SaHsfB2a	45–138	203–239	313–317 (KRART)		304–310 (KLFGFQL)	
SaHsfB2b	21–114	170–206	268–272 (KRVKR)		259–265 (KLFGVSI)	
SaHsfB2c	17–110	191–227	290–294 (KRRKK)		281–287 (MLFGVSI)	
SaHsfB2d	27–120	202–238	320–324 (KRARG)		311–317 (RLFGFQL)	
SaHsfB2e	19–112	177–213				
SaHsfB2f	41–134	229–265	355–359 (KRARE)		346–352 (SLFGYQL)	
SaHsfB4a	32–125	206–242	322-325 (SSSG)		312–318 (RLFGVPL)	336–338 (NLM)
SaHsfB4b	49–146	202–238	286–289 (KRFH)		276–282 (KLFGVSI)	
SaHsfC1a	8–102	127–170	196–201 (DKRRRM)			
SaHsfC1b	7–101	113–156	182–187 (NKRRRL)			

Note: DBD—DNA-binding domain; HR-A/B—oligomerization domain (OD); NLS—nuclear localization signal; AHA—activator motif; RD—repression domain; NES—nuclear export signal.

**Table 4:** Motifs identified by MEME tools.

Motif	Width	Reference Sequences
1	29	QRAAPPPFELTKTYQEMDDPSTDGIVSWSS
2	41	GNSFVVWDPEFARDLLPKYFKHNNFSSFVRQLNTYGFRKV
3	6	EGDCCC
4	29	DPDRWEFANEGFLRGZKHLLKTIKRRKPI
5	15	ACVEVGKYGLEEEVE
6	65	LKPDKNVLMEJVKLKQZQQSSDKQLLMDRLQGMEQRQQQMMSFLAKAVQNPGFLSQFVQQQA
7	10	HHPHLSGRSC
8	6	SPPPPP
9	6	NKKRRL
10	16	TGVNDVFMWEQYLTEHP
11	21	DKNQNINNITDQMGLLTSSAK
12	22	GGQIVKYQPFLDDMPTFFRNMM
13	22	FDIENIPPEHENTDGSAYDDVM
14	11	IASEPIPRPME
15	51	RAGLESDSHNNVDVQQPESVARIEESNLDSVARIEESNLDSVNZKLWDELLAGNLVJDNDDD
16	26	QLLGDIPEAEDLEGQPSDWEEEDLQ
17	18	HWEKIJYQIGRECGEDMF
18	6	HHHHNN
19	41	RAELMEENERLKKEINTQLTSELSHMKLCTNIYSMMSNYNP
20	8	KRFHNNGH
21	14	PKLFGVQIGSKRAR
22	41	QDPGNPSMESKLQLDLLQSEKYLEEQGGSSGGNAGAMDEKEC
23	17	VGCGSSNSSQAESMMNP
24	9	DLLQLQQPG
25	14	LLAAVVETQAIQAA

Note: Motif numbers corresponded to the motifs in Figure 3.

## Supplementary Materials

Supplementary materials can be found at http://www.mdpi.com/1422-0067/19/4/1216/s1.

## Abbreviations

aa	amino acid
ABA	abscisic acid
bp	base pair
kDa	kilodalton
pI	isoelectric points
qRT-PCR	quantitative real-time polymerase chain reaction
SG-U	synthetic-galactose-uracil
X-α-gal	5-bromo-4-chloro-3-indoxyl-α-d-galacto-pyranoside
SD	synthetic dropout nutrient medium
SD/Trp^−^	SD medium lacking tryptophan
SD/Trp^−^His^−^	SD medium lacking tryptophan and histidine
